# Celiac artery compression: Dunbar syndrome

**DOI:** 10.1590/1677-5449.009418

**Published:** 2019-05-23

**Authors:** Giovanna Mezzalira Santos, Luiz Marcelo Aiello Viarengo, Marcos Danillo Peixoto Oliveira

**Affiliations:** 1 Universidade de Taubaté – UNITAU, Faculdade de Medicina, Taubaté, SP, Brasil.; 2 Hospital Regional do Vale do Paraíba, Departamento de Cardiologia Intervencionista, Taubaté, SP, Brasil.

**Keywords:** Dunbar syndrome, celiac artery compression syndrome, median arcuate ligament syndrome, síndrome de Dunbar, síndrome da compressão da artéria celíaca, síndrome do ligamento arqueado mediano

## Abstract

Celiac artery compression syndrome, also referred to as median arcuate ligament syndrome, celiac axis syndrome or Dunbar syndrome is a rare disorder consequent to extrinsic compression of the celiac trunk by the median arcuate ligament. Doppler ultrasound, multi-slice computed tomography angiography, magnetic resonance angiography, or invasive selective angiography can identify stenosis of the initial segment of the celiac artery and confirm diagnosis. Treatment options include open surgical or videolaparoscopic section of the median arcuate ligament and the fibers of the celiac plexus, or percutaneous transluminal angioplasty via an endovascular approach. We report herein an interesting case of a 38-year-old woman diagnosed with this rare condition and successfully treated with the surgical strategy.

## Resumo

A síndrome da compressão da artéria celíaca, também denominada síndrome do ligamento arqueado mediano, síndrome do eixo celíaco ou síndrome de Dunbar, é uma doença rara causada pela compressão extrínseca do tronco celíaco pelo ligamento arqueado mediano. Ultrassonografia Doppler, angiotomografia computadorizada, angiorressonância magnética ou angiografia seletiva invasiva conseguem identificar a estenose do segmento inicial da artéria celíaca e confirmar o diagnóstico. As opções de tratamento incluem secção videolaparoscópica ou laparotômica (a céu aberto) do ligamento arqueado mediano e das fibras do plexo celíaco, assim como angioplastia transluminal percutânea. Relatamos o interessante caso de uma mulher de 38 anos de idade diagnosticada com essa rara condição e adequadamente tratada pela estratégia cirúrgica.

## INTRODUCTION

First described by Harjola^1^ in 1963 and Dunbar et al.^2^ in 1965, celiac artery compression syndrome (CACS), also referred to as median arcuate ligament syndrome (MALS), celiac axis syndrome (CAS), and Dunbar syndrome is a rare disorder consequent to extrinsic compression of the celiac trunk by the median arcuate ligament.^3^

CACS is more prevalent in children and adolescents and is associated with specific symptoms, mainly during expiration. Symptoms include the classic triad of mesenteric ischemia: postprandial abdominal pain, nausea, and vomiting and subsequently weight loss.^4^^,^ 5 Pain is linked to compression of the celiac trunk (and probably also the celiac plexus) at the level of the diaphragm, due to insertion of the arcuate ligament at a lower level.^3^

Once the syndrome is suspected, Doppler ultrasound (US), multi-slice computed tomography angiography (MSCTA), magnetic resonance angiography, or invasive selective angiography can identify stenosis of the initial segment of the celiac artery and confirm diagnosis. Treatment options include open surgical or videolaparoscopic section of the median arcuate ligament and the fibers of the celiac plexus, or percutaneous transluminal angioplasty via an endovascular approach.^4^^,^ 5

We report herein an interesting case of a 38-year-old woman diagnosed with this rare condition and successfully treated using the surgical strategy.

## CASE DESCRIPTION

**Figure 1:**
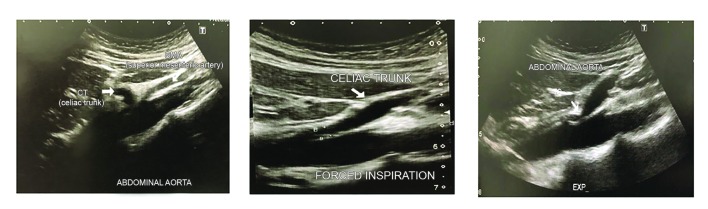
US images showing end-expiratory (right panel) compression of the CT. Left panel, at rest; central panel, with deep inspiration; right panel, with deep expiration. CT = celiac trunk; SMA = superior mesenteric artery; EXP = expiration.

**Figure 2:**
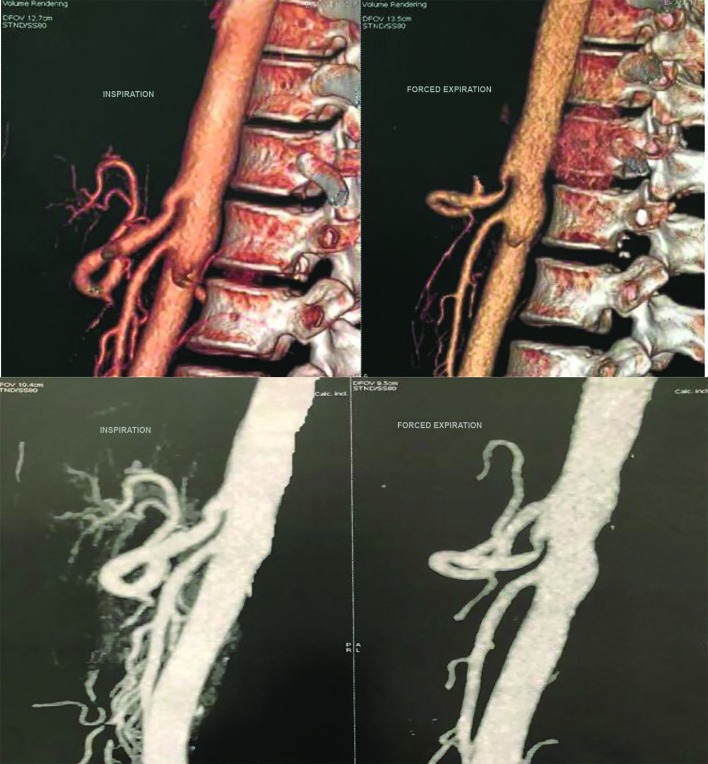
Multi-slice computed angiotomography with respiratory maneuvers - inspiration (left panels) and expiration (right panels). The typical hook-like downward stenosis of the celiac artery is due to the extrinsic compression by the arcuate ligament, especially at deep end expiration (right superior and inferior panels).

**Figure 3:**
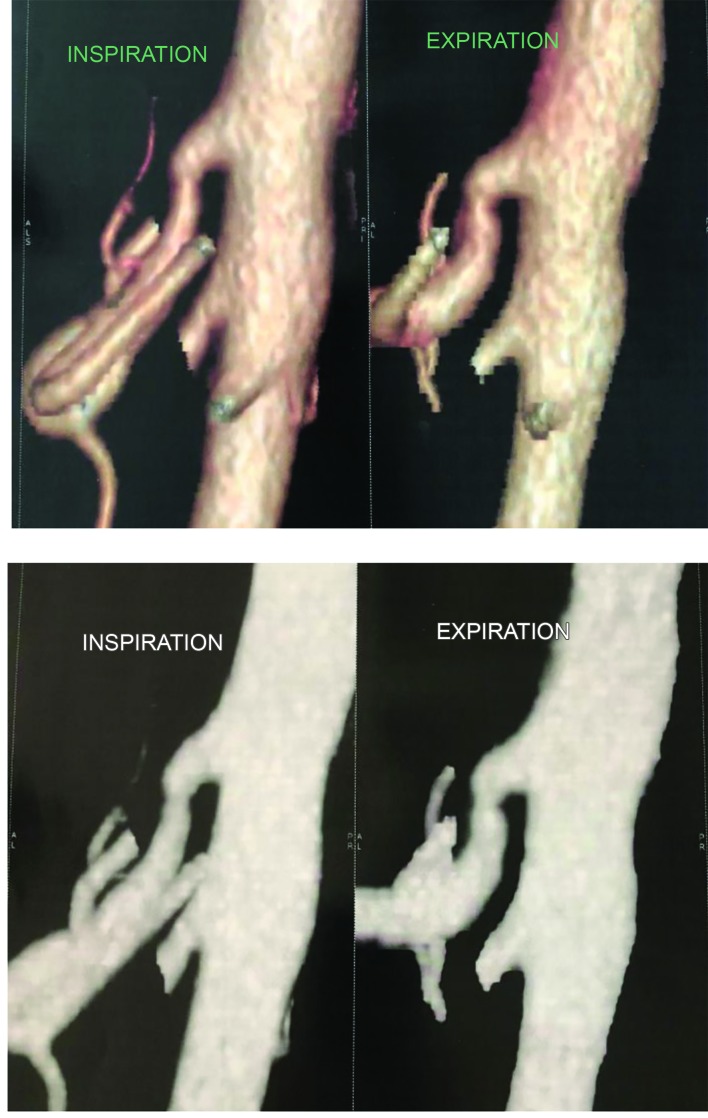
Postoperative multi-slice computed angiotomography with respiratory maneuvers - inspiration (left panels) and expiration (right panels) showing decompression of the celiac trunk.

An active, Caucasian, otherwise healthy 38-year-old-woman was found to have a high mesogastric murmur, exacerbated during deep expiration. The remainder of her clinical examination was unremarkable, except for the presence of superficial varicose veins in her legs. Her complaints were post-prandial abdominal pain, dyspepsia, and post-exertional fatigue. Initial Doppler US (Figure 1) and subsequent MSCTA (Figure 2) suggested and confirmed, respectively, extrinsic celiac artery compression by the median arcuate ligament, compatible with CACS. In view of her symptoms, which were causing significant food intake restrictions and weight loss, surgical (laparotomic) treatment of the condition was proposed to the patient. The procedure was undertaken uneventfully and successfully, with section of the median arcuate ligament and the fibers of the celiac plexus. Three months later, the patient is totally free from any symptoms and a follow-up MSCTA (Figure 3) showed decompression of the celiac trunk.

## DISCUSSION

CACS, MALS, CAS or Dunbar syndrome is a rare vascular compression syndrome, characterized by postprandial intestinal angina caused by impaired blood supply from the celiac artery to the gastrointestinal tract. The median arcuate ligament is located at the T12-L1 level and bridges the crura of the diaphragm, just anterior to the aorta. The celiac plexus is located between the arcuate ligament and the celiac trunk in up to 25% of normal individuals. Compression of the celiac trunk is, among other causes, secondary to diaphragm descensus after a period of accelerated growth in adolescents.^3^^-^ 5

The condition has a 3:1 female to male ratio and the classic patient is a woman aged between 18 and 30 years.^3^^-^ 5 Our patient was a 38 year-old woman and abdominal pain was her main symptom.

CACS is a rare entity, which is diagnosed in only 2 of 100,000 patients with ambiguous upper abdominal pain.^6^ The incidence of this disease is not known and typical symptoms are chronic or recurring epigastric pain (especially post-prandial), nausea, vomiting, diarrhea, weight loss, epigastric bloating, and reduced appetite. Since these symptoms may be caused by other diseases, like esophagitis, pancreatitis, cholelithiasis, and food intolerance, CACS is a diagnosis of exclusion.^3^^-^ 5

The classic manifestation of abdominal angina is seen in about 40% of patients. Two theories have been suggested to explain symptoms: compression of the mesenteric artery with mesenteric ischemia and splanchnic vasoconstriction due to stimulation of the celiac ganglion and celiac plexus.^3^^-^ 5 Pain seems to be related to mechanical irritation of the celiac plexus nerve fibers. As in the present case, an epigastric bruit may be detected on clinical examination.

CACS can be investigated with Doppler US, MSCTA, magnetic resonance angiography, and selective invasive angiography. Doppler US has high sensitivity for diagnosis and has been proposed as the modality of choice, although the gold standard diagnostic method is still selective angiography, which should be performed during both inspiration and expiration. However, the introduction of MSCTA has enabled acquisition of thinner images, providing increased resolution, improved lesion detection, and excellent multiplanar reconstructions.^7^ Since stenosis is respiratory-dependent and becomes more obvious with deep expiration, respiratory maneuvers are needed for diagnosis. The classic hook-like downward displacement followed by a dilatation of the celiac artery is a typical finding.^3^^-^ 7 The present case was diagnosed using Doppler US and MSCTA.

Treatment modalities include endovascular (percutaneous transluminal angioplasty with stent implantation) and open (laparotomic) or videolaparoscopic surgical procedures.^4^^,^ 5 The first option does not always solve the problem of extrinsic compression and surgical intervention is sometimes needed.^3^^-^ 7 Symptomatic patients with confirmed CACS will benefit more from surgical treatments, based on direct visualization and division of the arcuate ligament to achieve decompression of the celiac artery. In some cases, reconstruction of the artery or interposition of a graft is necessary. Decompression by minimally invasive surgery should be the treatment of choice. Laparoscopic techniques and, more recently, robotically-assisted decompression have been reported as safe treatment modalities.^3^^-^ 7

In the present case, the choice of technique (open surgery) was based on the surgical team’s experience, whose previous cases treated by laparoscopy had not been fully decompressed, especially when there was a strong fibrotic band and the celiac ganglion was involved in the compression, as in this case. In our opinion, laparoscopic surgery is much more time-consuming and generally incomplete, most likely because of fear of accidentally causing an arterial injury.

In conclusion, CACS is a rare and uncommon cause of postprandial abdominal pain and should be kept in mind after eliminating all other commonly encountered causes. Open surgery is an effective and safe treatment option in selected symptomatic patients, with good long-term results.
